# Impacts of stress and sex hormones on dopamine neurotransmission in the adolescent brain

**DOI:** 10.1007/s00213-013-3415-z

**Published:** 2014-01-31

**Authors:** Duncan Sinclair, Tertia D Purves-Tyson, Katherine M Allen, Cynthia Shannon Weickert

**Affiliations:** 1 Schizophrenia Research Institute, Sydney, Australia; 2 Schizophrenia Research Laboratory, Neuroscience Research Australia, Sydney, Australia; 3 Macquarie Group Chair of Schizophrenia Research, Neuroscience Research Australia, Barker Street, Randwick, NSW 2031 Australia; 4 School of Psychiatry, University of New South Wales, Sydney, Australia; 5 Neuropsychiatric Signaling Program, Center for Neurobiology and Behavior, Department of Psychiatry, University of Pennsylvania, Philadelphia, PA USA; 6 School of Medical Sciences, University of New South Wales, Sydney, Australia

**Keywords:** Adolescence, Dopamine, Schizophrenia, Development, Stress, Testosterone, Estrogen, Glucocorticoids

## Abstract

**Rationale:**

Adolescence is a developmental period of complex neurobiological change and heightened vulnerability to psychiatric illness. As a result, understanding factors such as sex and stress hormones which drive brain changes in adolescence, and how these factors may influence key neurotransmitter systems implicated in psychiatric illness, is paramount.

**Objectives:**

In this review, we outline the impact of sex and stress hormones at adolescence on dopamine neurotransmission, a signaling pathway which is critical to healthy brain function and has been implicated in psychiatric illness. We review normative developmental changes in dopamine, sex hormone, and stress hormone signaling during adolescence and throughout postnatal life, then highlight the interaction of sex and stress hormones and review their impacts on dopamine neurotransmission in the adolescent brain.

**Results and conclusions:**

Adolescence is a time of increased responsiveness to sex and stress hormones, during which the maturing dopaminergic neural circuitry is profoundly influenced by these factors. Testosterone, estrogen, and glucocorticoids interact with each other and have distinct, brain region-specific impacts on dopamine neurotransmission in the adolescent brain, shaping brain maturation and cognitive function in adolescence and adulthood. Some effects of stress/sex hormones on cortical and subcortical dopamine parameters bear similarities with dopaminergic abnormalities seen in schizophrenia, suggesting a possible role for sex/stress hormones at adolescence in influencing risk for psychiatric illness via modulation of dopamine neurotransmission. Stress and sex hormones may prove useful targets in future strategies for modifying risk for psychiatric illness.

## Introduction

Human adolescence is a developmental period of change for the body and the brain. As the adolescent brain matures, molecules and cells of the brain change to enable adaptive behaviors, which facilitate teenagers’ development of independence, forward planning, and resilience to psychosocial stress. Importantly, brain changes underpinning these behavioral adaptations occur in the context of puberty and sexual maturation. Healthy brain development, therefore, is fostered by appropriate interactions between maturing brain systems, sex hormones, and stress hormones during adolescence and results in acquisition of cognitive processes such as inhibitory control, long-term planning, and abstract problem solving, which are required to cope with the diverse cognitive and social demands that adults face.

Abnormal brain development at adolescence may play an important role in the emergence of psychopathology. Adolescence is a critical period for manifestation of mental illness, in particular schizophrenia, but also bipolar illness and depression (Christie et al. 1988; Hafner 2003; Hankin et al. 1998). Adolescent males are more prone to schizophrenia (Abel et al. 2010; Hafner 2003; Hafner et al. 1998), and young people with decreased tolerance of normal stress during adolescence are at an increased risk of transition to psychotic mental illness (Yung et al. 2005). Additionally, teenage girls face a higher risk for depression than they did at a younger age and a greater risk for depression than their male counterparts (Hankin et al. 1998). Adolescence is also an era of increased experimentation with, and abuse of, illicit drugs. In this period, first drug use typically occurs (average age 17.6; USA 2009), illicit drug use peaks (ages 18–20), and gender differences in drug use emerge (SAMSHA 2010). These observations highlight the potential for adolescent development to modify an individual’s mental health and emphasize the relevance of gender, sex hormones, and stress in shaping their thoughts and behavior.

This review focuses on adolescent changes in the dopamine signaling pathway, a key neurotransmitter pathway which has been heavily implicated in the pathophysiology of schizophrenia (Abi-Dargham et al. 2000; Abi-Dargham et al. 2011; Akil et al. 1999; Bertolino et al. 2009; Howes et al. 2011; Howes et al. 2009; Laruelle and Abi-Dargham 1999; Meyer-Lindenberg et al. 2002) (summarized in Fig. 3). Dopaminergic signaling is critical to the adolescent development of working memory (Goldman-Rakic 1996), which is a cardinal cognitive process vital to reasoning and judgment. Working memory dysfunction is a hallmark of schizophrenia and can also be evident in depression and bipolar illness (Goldman-Rakic 1994; Meyer-Lindenberg et al. 2005). Adolescent changes in dopamine signaling occur in the prefrontal cortex (PFC), which is responsible for working memory, and in subcortical regions, which are involved with probabilistic learning and reward (Morris et al. 2012; Weickert et al. 2009b). In these brain regions, we will outline the pattern of change in key dopamine-related parameters, focusing on adolescence, and explore how they are influenced both by sex and stress hormones.

### Defining adolescence

Adolescence is regarded as “the gradual period of transition from childhood to adulthood” (Spear 2000), which encompasses puberty, the period during which sexual maturity is attained [for detailed reviews, see also Schneider (2008, 2013) and Sisk and Foster (2004)]. This review will consider neurobiological changes during adolescence in humans and animal models, primarily rodents and primates. Adolescent rodents model some aspects of human adolescent behaviors, such as greater social interaction (Primus and Kellogg 1989) and increased novelty-seeking behavior (Adriani et al. 2002) compared to adults, and are commonly employed. In rodents, adolescence can be considered to extend from the time just after weaning until after the completion of sexual maturity (Schneider 2008; 2013). Therefore, at its broadest definition, adolescence in female rats has been defined as between postnatal day (PND) 22 and 60, and males from PND28 to 70 (Schneider 2013), with similar definitions for mice (Adriani et al. 2002). However, adolescence can also be defined using different physiological parameters, such as physical features, sex steroid levels, the attainment of sexual maturity, and reproductive capability (Hill et al. 2012; Lewis et al. 2002; Naninck et al. 2011; Saksena and Lau 1979; Walker et al. 2012). This has led to large variations in what different research groups refer to as adolescence and difficulties in comparing studies. In addition, many studies provide animal weight only, which is not a reliable indicator of exact age (McCutcheon and Marinelli 2009). Nonhuman primates may be considered to model human adolescence more accurately than rodents, since, like humans, they have an extended adolescence lasting months to years (Schwandt et al. 2007). They also have more protracted postnatal development in general, a more similar endocrine system, more complex social organization, and have more evolved cerebral cortices than rodents (Barr et al. 2004). Additionally, more extensive cognitive and social tests can be administered to both primates and humans than to rodents (Plant 1996). In the rhesus macaque, adolescence is between 2 and 4 years (Lewis 1997; Schwandt et al. 2007), whereas in the marmoset, adolescence starts at 21 weeks and extends to 12 months (Pryce et al. 2004). Human adolescence has been defined as between age 10 and 17 in girls and age 12 to 18 in boys (Falkner and Tanner 1986), although others consider that adolescence extends until age 25 (Baumrind 1987). Overall, care must be taken in performing, interpreting, and comparing studies of adolescence in humans and animal models, bearing in mind the likelihood of adolescence-related differences between genders and species, and the challenges associated with reproducibly defining adolescence from one study to the next.

## Dopamine signaling during adolescence

### Developmental changes in dopamine signaling

**Fig. 1:**
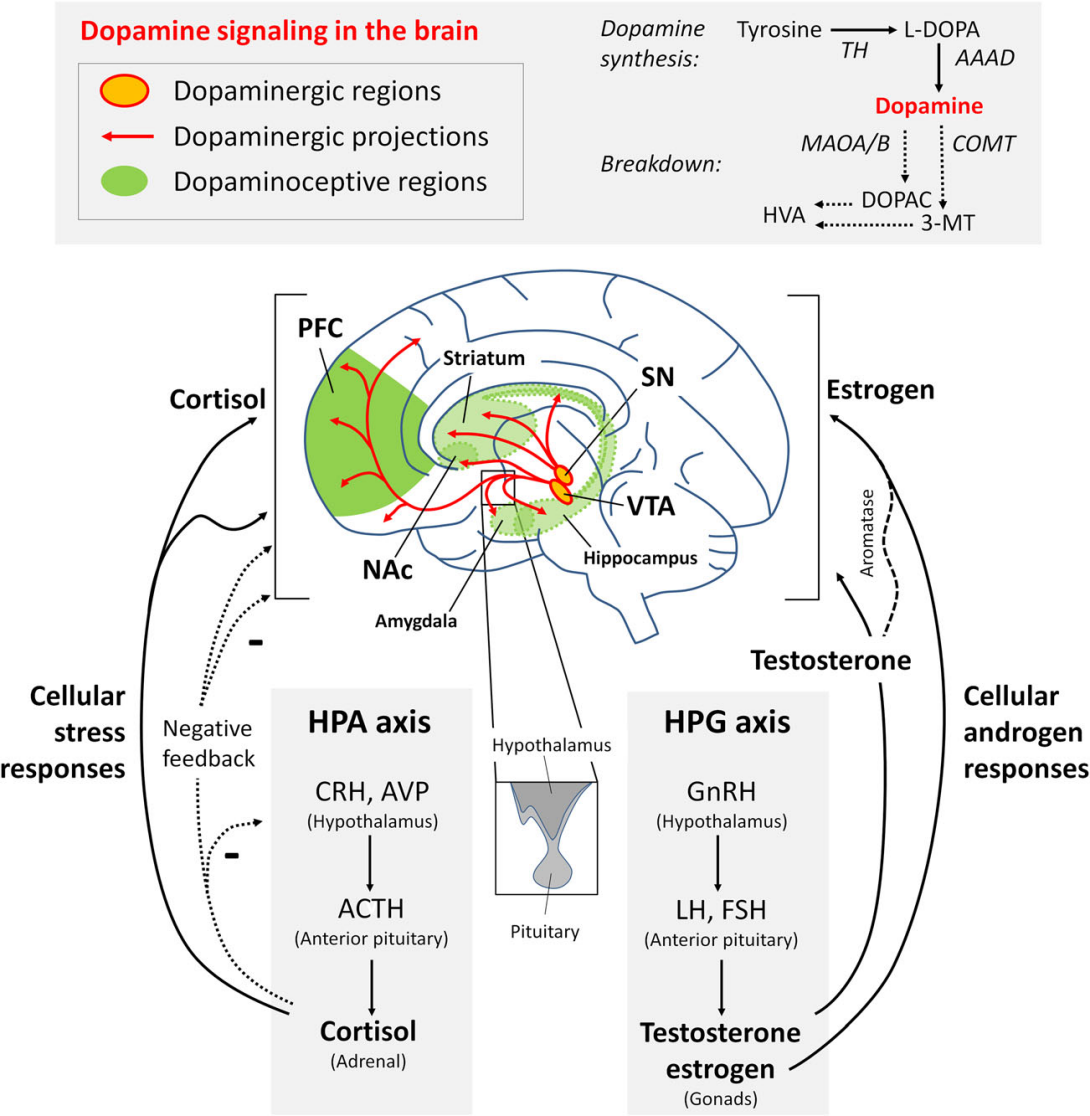
Dopamine signaling in the brain, and the production of glucocorticoid, androgenic, and estrogenic steroids by the HPA and HPG axes. *Darker* and *lighter shading* indicate superficial and deep structures, respectively. *Abbreviations*: *HPA* hypothalamic-pituitary-gonadal, *HPA* hypothalamic-pituitary-adrenal, *PFC* prefrontal cortex, *NAc* nucleus accumbens, *VTA* ventral tegmental area, *SN* substantia nigra, *CRH* corticotropin-releasing hormone, *AVP* arginine vasopressin, *ACTH* adrenocorticotropic hormone, *GnRH* gonadotropin-releasing hormone, *LH* luteinizing hormone, *FSH* follicle-stimulating hormone, *TH* tyrosine hydroxylase, *L*-*DOPA* L-3,4-dihydroxyphenylalanine, *AAAD* aromatic l-amino acid decarboxylase, *MAOA*/*B* monoamine oxidase A/B, *COMT* catechol-*O*-methyl transferase, *DOPAC* dihydroxyphenylacetic acid, *3*-*MT* 3-methoxytyramine, *HVA* homovanillic acid

**Fig. 2:**
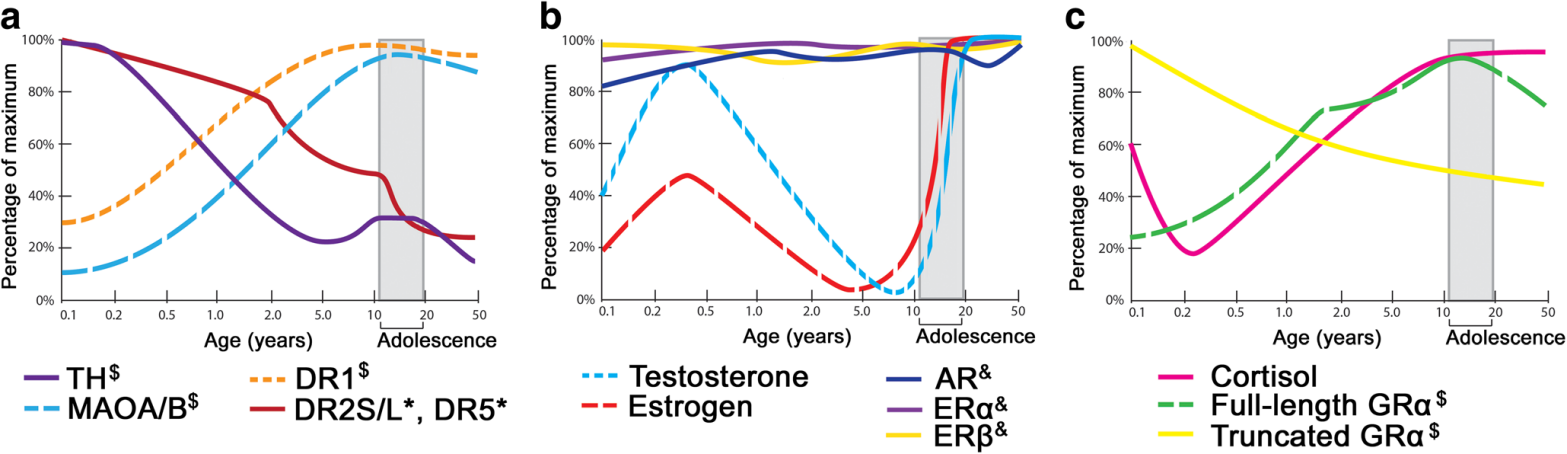
**a**–**c** Developmental changes in expression of mRNA transcripts and proteins in the dopamine and stress signaling pathways in the human PFC. *Dollar sign*, molecules whose protein abundance is plotted; *asterisk*, molecules whose mRNA transcript abundance (confirmed by qPCR) is plotted; *ampersand*, unpublished microarray data (Weickert et al., unpublished). *Abbreviations*: *TH* tyrosine hydroxylase, *MAOA*/*B* monoamine oxidase A/B, *DR* dopamine receptor, *AR* androgen receptor, *ER* estrogen receptor, *GR* glucocorticoid receptor. Data drawn from Gunnar et al. (2009), Kiess et al. (1995), Naninck et al. (2011), Rothmond et al. (2012), Sinclair et al. (2011), and Sippell et al. (1980)

The midbrain dopaminergic neural circuitry consists of dopaminergic neurons in the substantia nigra (SN) and ventral tegmental area (VTA), and dopamine-responsive neurons in the PFC, nucleus accumbens (NAc), hippocampus, entorhinal cortex, amygdala, and striatum (Fig. 1). Here, we briefly review changes in the dopamine system over adolescence and refer the reader to a more extensive review by Wahlstrom et al. (2010). Across the lifespan in the human and rodent PFC, dynamic changes take place in the expression of the most abundant dopamine receptors (DRs), which include DR1 and DR5 (both “DR1-like”) and DR2 and DR4 (both “DR2-like”). Human cortical DR1 mRNA and protein do not attain adult levels until late adolescence/early adulthood (Fig. 2) (Rothmond et al. 2012; Weickert et al. 2007). In humans, this postnatal increase in DR1 is unique among dopamine mRNAs, as the transcripts encoding DR5 and DR2 both decrease dramatically shortly after birth and reach steady levels just after toddlerhood (Fig. 2), whereas DR4 appears to be more evenly expressed across age (Rothmond et al. 2012; Weickert et al. 2007). In the rat orbitofrontal cortex, adolescent increases in DR1 expression may contribute to adolescent changes in associative learning, which can be modulated by DR1 agonism or DR2 antagonism (Garske et al. 2013). Since DR1 is found abundantly on postsynaptic dendrites of pyramidal neurons and stimulates G protein-coupled receptors, this may result in increased excitatory potential for dopamine at maturation which can combine with glutamatergic signaling to drive neurons into a more active “up-state” (O'Donnell 2010). Further, work in nonhuman primates indicates that synaptic contact of dopamine onto dendrites is increased dramatically during postnatal life (Lambe et al. 2000). In rhesus macaques, the axon length and varicosity density of neurons immunoreactive for tyrosine hydroxylase (TH), the dopamine synthetic enzyme, peaks at adolescence (2–3 years of age) in the middle cortical layers of the prefrontal cortex and then declines to stable adult levels (Rosenberg and Lewis 1995). While this suggests increased dopamine influence over pyramidal neurons during adolescence in monkeys, in human PFC, there is a dramatic decrease in overall levels of TH (Fig. 2) (Rothmond et al. 2012) and decreases in DR2 and DR5, suggesting that the global cortical action of dopamine in humans may attenuate over time. Alternatively, these global decreases may occur as the action of dopamine at adolescence become more focal, both anatomically (at the postsynaptic dendrite) and temporally. This interpretation is supported by the fact that proteins that can break down dopamine are either at their highest levels in adolescence or adulthood in the human PFC (MAOA and MAOB; Fig. 2) or achieve increased activity states at that time (COMT) (Rothmond et al. 2012; Tunbridge et al. 2007). If increased degradation of dopamine were to occur at the synapse during adolescence, this may reduce the time frame during which dopamine can bind to its receptors, resulting in a more temporally focused action of dopamine during adolescence.

Another consideration is that dopamine’s actions at cortical interneurons shifts during adolescence, whereby DR2 stimulation that was previously inhibitory becomes excitatory (O’Donnell 2010). In rats at PND14–35, inhibitory fast spiking interneurons in the PFC are unresponsive to DR2 activation; however, in rats at PND50, DR2 activation is excitatory in these neurons (Tseng and O’Donnell 2007), indicating that at some point between PND35 and 50 (i.e., in adolescence), the switch has occurred. Interestingly, DR1 and DR2 binding densities in the rat cortex reach a peak at PND40 and then gradually decrease to a nadir at PND100 (Andersen et al. 2000). In rodents, DR4 binding increases between PND7 and PND21 and thereafter, as in humans, remains relatively unchanged (Tarazi and Baldessarini 2000). Dopamine plays a critical role in tuning pyramidal neurons involved in working memory either directly, or indirectly through inhibitory neurons, and the correct amount of dopamine (not too little and not too much) is required for optimal signal to noise and accurate information processing (Goldman-Rakic 1996), indicating that changes in the control of dopamine neurotransmission at adolescence contribute to the attainment of mature functional ability of the human PFC. In rats, cortical output neurons to the NAc have been shown to express higher DR1 levels during adolescence (PND44) compared to juvenile (PND27) and older (PND105) ages, and this has been linked to increased sensitivity of adolescents to addictive behaviors (Brenhouse et al. 2008). Changes in the regulation of glutamatergic output from the PFC to the NAc by dopamine may therefore contribute to drug-seeking behavior during human adolescence. Overall, these findings highlight the dynamic changes occurring in dopaminergic neurotransmission in the cortex at adolescence and suggest that such changes may play a role in the functional maturation of the cortex.

Dopaminergic signaling also changes during subcortical maturation, although this appears to be temporally different to the maturation of the cortex. Dopamine receptors are mostly localized to postsynaptic sites in the striatum [caudate putamen (CPu) and NAc], although DR2 is also found presynaptically. In rats, striatal increases in DR1 and DR2 binding occur throughout early postnatal life, to peak at PND28 before decreasing at PND35 and remaining constant thereafter (Tarazi and Baldessarini 2000). Interestingly, sex differences in the adolescent developmental profiles of DR1 and DR2 in the rat striatum were observed in another study, where male DR1 and DR2 densities peaked at around PND40 before decreasing rapidly until PND80, whereas females exhibited more constant levels over development (Andersen et al. 1997). This observation led the authors to hypothesize that gender differences in developmental dopamine receptor expression may contribute to the increased prevalence of dopaminergic disorders such as ADHD in males (Andersen and Teicher 2000). DR4 mRNA levels in the rodent striatum reach their highest levels at PND3 (Nair and Mishra 1995), but DR4 binding (which is low relative to DR1 and DR2) peaks around PND28, like DR1 and DR2 (Tarazi and Baldessarini 2000). Dopamine firing rates in the rat midbrain are low post-weaning, increase and peak at PND45, and then decline into adulthood (McCutcheon and Marinelli 2009). In human development, one study has indicated that striatal DR1 and DR2 binding peak at ~3 years of age and decrease gradually throughout adulthood (Seeman et al. 1987); however, only three brains from individuals between 10 and 20 years of age were analyzed, so the adolescent period is difficult to assess. In humans, nigrostriatal dopamine neurons exhibit the highest levels of TH activity in childhood, and this decreases exponentially over the next 30 years (Segawa 2000). These developmental trajectories of dopamine-related molecules have led to the hypothesis that subcortical dopamine targets mature before those in the cortex. It is speculated that this results in changes to impulsivity and reward-seeking behaviors which precede mature cortex-driven decision making (Geier and Luna 2009), leading to increased risk for drug addiction, dangerous reward-seeking behavior, and possibly psychiatric illness. Importantly, the spatial and temporal sensitivity of the cortex and the striatum to dopamine may be shaped by sex and stress hormones at adolescence in such a way as to modulate the timing of cortical and subcortical dopamine-related maturation.

## Sex and stress hormones during adolescence

Molecular, cellular, and functional changes in the dopamine neurotransmitter system at adolescence occur in the context of dramatic changes in sex and stress hormones. Understanding the changes in sex and stress hormone signaling at adolescence is key to identifying the impacts of these hormones on cortical and subcortical dopaminergic neurotransmission at adolescence.

### Sex hormones

The major sex steroids—androgens and estrogens—play a fundamental role in the development and differentiation of the adolescent brain. Circulating sex hormones are produced by the gonads (Fig. 1) and readily cross the blood brain barrier. They affect multiple brain regions beyond those involved in sexual and reproductive behaviors, including the dorsolateral prefrontal cortex (DLPFC) (Bailey et al. 2011; McEwen 2001). An understanding of sex hormone signaling in the adolescent brain, particularly in the brain regions underlying social interaction, reward, cognition, and working memory, is vital to understanding gender dimorphism in the brain and may shed light on sex differences in cognition, mood, and susceptibility to a range of mental disorders.

Gonadal sex steroid production changes dramatically across the lifespan and drives sexual differentiation of the brain in utero and during postnatal life (Phoenix et al. 1959; Schulz et al. 2009). In humans, testosterone (in males) and estrogens (in females) peak in the middle trimester of gestation, then drop in the perinatal period and peak a second time within the first 3 months of life (Fig. 2) (Auyeung et al. 2013; Naninck et al. 2011). These pre- and postnatal stages are thought of as periods of permanent “organizational” sexual differentiation of the brain (Arnold and Breedlove 1985; Phoenix et al. 1959). Sex steroid levels then remain low until adolescence, when they rise rapidly from the age of ~10 years reaching adult levels between the ages of 16 and 19 years (Fig. 2). Adolescence involves “activational” effects of sex hormones that stimulate circuits and behavioral patterns set up during pre- and early postnatal development (Arnold and Breedlove 1985; Vigil et al. 2011). Imaging data highlight that this period may also involve organizational effects of sex steroids, as brain size, structure, and wiring can change during adolescence (Lenroot and Giedd 2010; Schulz et al. 2009; Sisk and Zehr 2005). Thus, the organizational effects of sex steroids may occur early in life and during adolescence, while activational effects of sex steroids may occur during adolescence or adulthood.

**Fig. 3:**
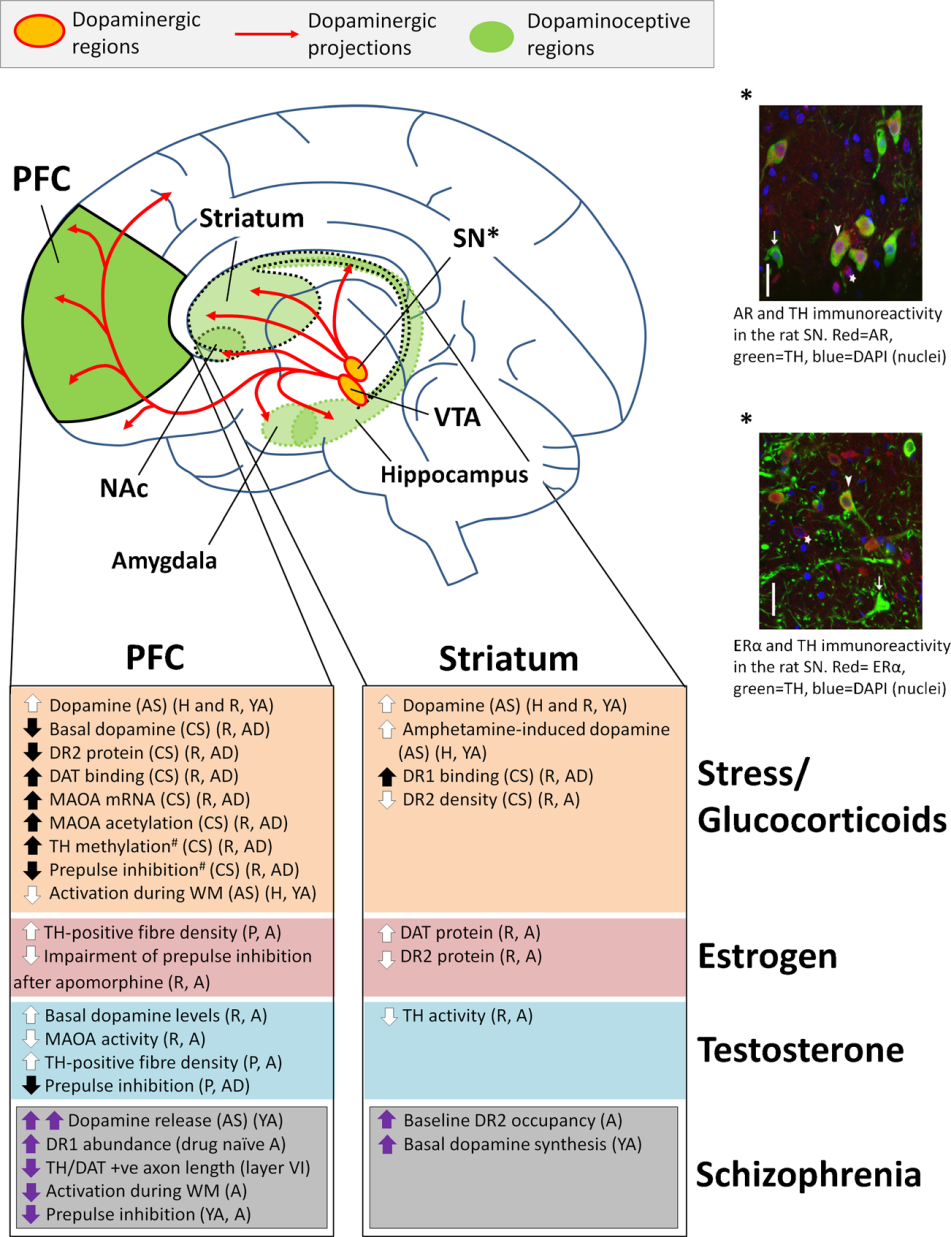
Summary of the effects of stress and sex hormones on dopaminergic signaling in the PFC and striatum. For comparison purposes, dopamine abnormalities found in schizophrenia are also presented. References and details of summarized studies are described in the text and Table 1. *Black arrows within shaded boxes* indicate findings in adolescents, *thick white arrows* indicate findings made in young adults or adults that have yet to be demonstrated in adolescents, and *thick purple arrows* indicate findings in schizophrenia. *Number sign*, stress effects seen in disrupted in schizophrenia 1 (DISC1) mutant mice but not wild-type mice in the same chronic isolation stress paradigm. For immunohistochemistry images, *white arrowheads* indicate cells immunoreactive for both steroid receptors and TH, *arrows* indicate cells immunoreactive for TH only, and *stars* indicate cells immunoreactive for steroid receptors only. *Scale bars* represent 40 μm. Images provided by Owens and Purves-Tyson (unpublished). *Abbreviations*: *PFC* prefrontal cortex, *NAc* nucleus accumbens, *SN* substantia nigra, *VTA* ventral tegmental area, *AS* acute stress, *CS* chronic stress, *H* human, *P* primate, *R* rodent, *AD* adolescent, *YA* young adult, *A* adult, *DR* dopamine receptor, *DAT* dopamine transporter, *MAOA* monoamine oxidase A, *TH* tyrosine hydroxylase, *WM* working memory

Estrogens and androgens (testosterone) exert their effects by binding to estrogen receptors (ERs) or androgen receptors (ARs). The actions of estrogen were thought to be mediated by a single estrogen receptor (ER, now termed ERα) (Greene et al. 1986; Horwitz and McGuire 1978), until a second estrogen receptor, ERβ (Kuiper et al. 1996), and yet a third estrogen receptor, G protein-coupled receptor 30 (GPR30) (Revankar et al. 2005; Thomas et al. 2005), were identified, establishing estrogen signaling as more complex than originally thought. We focus on ERα and ERβ which are transcription factors directly influencing gene expression, rather than GPR30 which is a G protein-coupled receptor associated with rapid signaling events. ERα and ERβ have distinct spatiotemporal patterns of expression in the brain (Kuiper et al. 1997), but are both expressed in human, primate, and rodent cortex (including the PFC) and midbrain during development and into adulthood (Gonzalez et al. 2007; Kuiper et al. 1997; Montague et al. 2008). Although ERα and ERβ were originally thought to be minimally expressed or absent from the nigrostriatal pathway in rodents (Laflamme et al. 1998; Mitra et al. 2003), our studies have identified mRNA for AR, ERβ, and ERα, and protein for AR and ERα, in dopamine neurons in the male rat SN (Purves-Tyson et al. 2012) (Fig. 3). A role for ERα in neuroprotection of the nigrostriatal pathway has also been identified (Al Sweidi et al. 2012; Bourque et al. 2009). AR receptors are robustly expressed in the neurons of the rat and primate cerebral cortices (Clark et al. 1988; DonCarlos et al. 2003; Nunez et al. 2003). Testosterone can bind AR directly, or can be converted via 5α-reduction to dihydrotestosterone (DHT), which binds AR with greater affinity (Celotti et al. 1992). DHT can be further converted to 5α androstane 3β, 17β diol (3β-diol) which has a high affinity for ERβ [reviewed in Handa et al. (2008)]. Testosterone can also act indirectly at ER, following its aromatization to estradiol, adding further complexity to mechanisms of action of testosterone.

Responsiveness to the sex steroids during adolescence, and across the lifespan, may be influenced by levels of ER, AR, aromatase, and 5α-reductase expression. Sex steroid receptor mRNA expression profiles in the DLPFC during human development have not been published, but unpublished microarray data, from a previously reported developmental microarray study (Weickert et al. 2009a), suggests that prefrontal cortical ERα, ERβ, and AR levels may not change appreciably across the lifespan (Fig. 2). In contrast, in the temporal cortex, ERα and ERβ protein increases gradually from birth to adulthood (Gonzalez et al. 2007). In rodents, cortical ERα mRNA decreases and cortical ERβ mRNA increases over early postnatal development (PND4–28) (Westberry and Wilson 2012), implying potentially a greater contribution to adolescent brain development by ERβ. Aromatase has been detected in the human frontal cortex (Stoffel-Wagner et al. 1999), and higher levels of aromatase mRNA have been identified in the temporal cortex of adults than in children (Stoffel-Wagner et al. 1998). A longitudinal neuroimaging study has mapped male-specific changes in human cortical maturation during adolescence and reported that a functional allele of the AR gene may modify this process (Raznahan et al. 2010), implying that testosterone signaling may contribute to cortical maturation during adolescence. Sex steroid receptor expression can be modulated by sex steroids themselves. Experimental manipulation of sex hormones modulates levels of sex steroid receptor expression in the SN of adolescent male rats (PND45–60) and the cortex of adult male and female mice (PND150–180), and this modulation depends on the receptor subtype, the steroid treatment, sex, and age of the animal (Kumar and Thakur 2004; Purves-Tyson et al. 2012; Thakur and Sharma 2007). Overall, expression of sex steroid receptors and conversion enzymes during adolescence suggests that the maturing adolescent cortex and midbrain are equipped to respond to sex steroids. Therefore, as a result of the pubertal surge in sex steroids, adolescence is likely to be characterized by increasing responsiveness to, and influence of, sex hormones, particularly in cortical regions such as the temporal cortex, where expression levels of sex steroid receptors and conversion enzymes are greater in adolescence than earlier in life. Consequently, the maturation and function of the brain may be profoundly influenced by sex hormones during adolescence.

### Stress hormones

Glucocorticoid stress hormone secretion represents the primary means by which humans, and other vertebrates, respond and adapt to stressful environmental stimuli throughout life. Glucocorticoids (corticosterone in rodents, cortisol in humans) are secreted by the adrenal glands, following activation of the hypothalamic-pituitary-adrenal (HPA) axis (Fig. 1) (de Kloet et al. 2005). Basal daytime glucocorticoid secretion across the human lifespan has an early peak at the time of birth, before dropping to a nadir around 3 months of age, rising rapidly until 3 years of age and more gradually thereafter until adulthood (Kiess et al. 1995; Sippell et al. 1980). Teenagers (aged 9–19) display increasing morning and daytime basal cortisol levels with increasing pubertal maturation (Adam 2006; Kiess et al. 1995; Netherton et al. 2004; Oskis et al. 2009; Shirtcliff et al. 2012). In rodents, unlike in humans (Kiess et al. 1995), young adolescents (PND33–43) display higher basal corticosterone levels than adults (PND60+) (Adriani and Laviola 2000; McCormick et al. 2008). These findings are important because the increase in glucocorticoids circulating during adolescence, relative to early life (and adulthood in rodents), means that they may have a heightened effect on brain maturation and function during this time.

The glucocorticoid receptor (GR) mediates the cellular effects of glucocorticoids in times of stress (Kitchener et al. 2004), enabling adaptation of neurons and neural circuits, and additionally (in the PFC, hippocampus, and hypothalamus) mediating negative feedback regulation of the HPA axis (Diorio et al. 1993; Mizoguchi et al. 2003; Weiser et al. 2011). GR is abundant in the dopamine projection regions such as the PFC, hippocampus, and striatum (Morimoto et al. 1996; Webster et al. 2002) and is expressed to a lesser degree in the SN and VTA (Morimoto et al. 1996). GR mRNA and full-length GRα protein found in neurons and glia of the human PFC change dynamically across the lifespan, reaching their lifetime peak levels in adolescence (15–18 years; Fig. 2) (Perlman et al. 2007; Sinclair et al. 2011). In the rodent hippocampus, greater GR mRNA expression has been reported in adulthood (PND60) than earlier (PND30) (Bohn et al. 1994), but in humans, hippocampal GR may not change with age (Perlman et al. 2007; Wang et al. 2013). These findings suggest that human adolescence may represent a period of increased prefrontal cortical stress responsiveness facilitated by increased cellular GR expression.

There is evidence that acute and chronic stress elicit greater HPA axis responses in adolescents that adults. Glucocorticoid secretion in response to physical and psychological stressors has been shown to increase as humans grow (ages 9–19) (Adam 2006; Gunnar et al. 2009; Stroud et al. 2009), in parallel with increasing basal cortisol levels. In rodents, studies have reported more prolonged glucocorticoid release in response to acute stress (restraint stress or ether odor) in early adolescent rats (PND25–28) relative to adults (PND65–77) (Romeo et al. 2006; Vazquez and Akil 1993), while others have described blunted corticosterone response to other stressors (forced novelty or open arm of the elevated plus maze) in adolescents (PND33–43) compared to adults (PND60+) (Adriani and Laviola 2000; McCormick et al. 2008). Greater HPA axis responsiveness in adolescents than adults suggests that the brain may be subject to higher levels, and greater fluctuation, of glucocorticoids following stress in adolescence than adulthood.

Stress during adolescence can impact brain development and result in maladaptive changes later in life, which impact behavior and stress responsiveness. Whereas adult rats (PND77) display habituation to chronic stress (Girotti et al. 2006), reflected in decreased glucocorticoid secretion with successive stress exposures, early adolescent rats (PND28) do not, but instead respond more strongly to repeated exposures and return to baseline more quickly (Romeo et al. 2006). The effects of adolescent chronic stress on later-life stress responsiveness may vary according to species, timing, and type of stress. In adolescent humans at age 16, chronic stressful experiences earlier in adolescence (between ages 12 and 15) have been associated with blunted responsiveness to psychosocial stress, while stressful experiences during childhood (between ages 6 and 11) have been associated with increased responsiveness to psychosocial stress (Bosch et al. 2012). Lasting impairment of normal structural and functional brain development arises as a consequence of adolescent exposure to chronic stress. Chronic, variable stress from PND28 to 56 results in an arrest of normal hippocampal neurogenesis and neuronal maturation, observable 4 days and 3 weeks after cessation of stress, respectively (Isgor et al. 2004; McCormick et al. 2010). These developmental impairments are accompanied by impaired spatial memory and decreased hippocampal GR expression (Isgor et al. 2004; McCormick et al. 2010). At a behavioral level, chronic glucocorticoid administration or stress during early adolescence (PND28–42) can increase aggression, decrease social interaction, and cause anxiety-like behaviors in adulthood (PND90+) (Cordero et al. 2012; Marquez et al. 2013; McCormick et al. 2008; Veenit et al. 2013; Vidal et al. 2007; Wright et al. 2008). Adolescent stress (at PND38–46) has been shown to elicit greater behavioral impairments (measured 3 weeks after stress cessation) than the same stress administered during adulthood (PND60–68) (Wright et al. 2012). Other reviews (e.g., Lupien et al. 2009) detail the effects of early life stress, which are beyond this review’s scope. Taken together, the changes in the HPA axis across the lifespan, particularly cortisol secretion and molecular expression of GR in the brain, suggest that the PFC is primed to be highly stress sensitive during adolescence and thus equipped to respond to the unique burden of stressors experienced in the transition to adulthood. This adolescent stress sensitivity may increase susceptibility to the deleterious effects of chronic stress, with the potential to interfere with normal brain maturation and contribute to adolescent-onset or later-life psychopathology.

### The interaction of sex and stress hormones at adolescence

The sex and stress hormone systems have been shown to interact with each other, resulting in reciprocal modulation of each other’s expression and function. These interactions are likely to be particularly important during adolescence, when the sex and stress signaling systems are highly engaged together, and the brain is undergoing final maturation.

Sex hormones can modulate stress hormone secretion, HPA axis function, and stress-induced PFC dysfunction. Female rats display increased basal levels of corticosterone relative to males during adolescence (PND33–48) (Martinez-Mota et al. 2011; McCormick et al. 2005) and in adulthood (PND150) (Weinstock et al. 1998), while male rats display lower stress-induced hormone secretion after puberty than before (Foilb et al. 2011; Romeo and McEwen 2006). These gender differences appear attributable to sex hormones, since gonadectomy decreases stress-induced glucocorticoid secretion in adult female rats (PND120) (Burgess and Handa 1992) and increases glucocorticoid secretion in adult males (Handa et al. 1994). This suggests that estrogen has a stimulatory effect on HPA axis function, while testosterone has an inhibitory effect mediated by AR (since it is mimicked by DHT) (Handa et al. 1994). However, testosterone’s effects could also be due to a DHT metabolite (5α-androstane 3β, 17β diol), which is a potent ERβ agonist (Handa et al. 2011). The stimulatory effects of estrogen on the HPA axis activity are also seen during the female rodent estrous cycle, with basal and stress-induced glucocorticoid secretion increasing with increasing circulating estrogen levels (Carey et al. 1995). These stimulatory effects are mediated by ERα, which impairs negative feedback regulation of rodent glucocorticoid secretion (Weiser and Handa 2009). Adolescent female rats (PND40–48) may be more susceptible to the deleterious long-term behavioral effects of stress than males (Wright et al. 2008), and in adult rats, estrogen contributes to impaired PFC function during stress (Shansky et al. 2004). Conversely, estrogen may protect adolescent females (PND33–48) from anxiety-like behaviors after social stress (McCormick et al. 2008).

The influence of sex hormones on HPA axis activity in humans is less clear. A number of studies have reported greater HPA axis reactivity after psychosocial stress in late adolescent/young adult males than females (aged 19–33) (Kirschbaum et al. 1999; Kirschbaum et al. 1992; Kudielka and Kirschbaum 2005; Uhart et al. 2006). However, others have revealed increased HPA axis reactivity in females than males early in adolescence (age 13) (Gunnar et al. 2009) or in adult females (18–50, mean age 21) after pharmacological stress (Uhart et al. 2006). Morning cortisol levels are higher in mid/postpubertal girls (mean age 13.7 years) than pre/early pubertal girls (mean age 9.9 years), but not changed between mid/post and pre/early pubertal boys (Netherton et al. 2004). In adult humans (mean age 24), as in rodents, HPA axis reactivity increases with increasing estrogen and progesterone levels during the female menstrual cycle (Kirschbaum et al. 1996). Thus, sex hormones modulate HPA axis function in both humans and rodents during adolescence. As a result, the ability of teenagers to respond to the stresses of adolescence may be impacted by their pubertal development. Furthermore, the opposing effects of testosterone and estrogen on HPA axis activity suggest that sex hormones may differentially modulate stress sensitivity in males and females, which could contribute to gender differences in the incidence and symptomatology of stress-related psychiatric illnesses such as depression and schizophrenia.

While sex hormones can influence stress hormone secretion and function, the reverse is also true. Glucocorticoids may impact the action of testosterone and estrogen in the brain by decreasing synthesis, and circulating levels, of both sex steroids. This has been demonstrated in adults (Bambino and Hsueh 1981; Bernton et al. 1995; Michael et al. 1993) and adolescents, with the emergence of puberty in male rats delayed by early adolescent stress (PND28–42) (Marquez et al. 2013). This suggests that stress may impact the development of the reproductive axis at adolescence, which in turn may influence sex steroid-induced brain maturation. Glucocorticoids may exert these effects by inducing the expression, and potentiating the activity, of estrogen sulfotransferase, which converts estrogen to a sulfonated form unable to bind the estrogen receptor (Gong et al. 2008). It is not known whether this phenomenon occurs in the brain; however, it could represent a mechanism through which glucocorticoids modulate cortical sensitivity to circulating sex hormones. There may be a range of consequences of the modulation of sex hormone signaling in the brain by stress/glucocorticoids. For example, reproductive drive may be balanced and fine-tuned during adolescence due to increased stress responsiveness. Since estrogen stimulates glucocorticoid secretion but glucocorticoids inhibit estrogen secretion, these effects may serve to balance each other. However, since testosterone inhibits glucocorticoid secretion and glucocorticoids inhibit testosterone secretion, these effects may augment each other, which may result in potentiation of either testosterone- or glucocorticoid-mediated signaling depending on context. Further study of the interaction of sex and stress hormones may identify mechanisms through which balance of the sex and stress axes is maintained, particularly when sex hormone levels and stress levels are changing dramatically at adolescence.

## Effects of sex steroids on dopamine signaling

Sex steroids have the capacity to modulate dopamine signaling and may play an important role in regulating some of the adolescent developmental changes in dopamine systems described earlier. Determining which of these changes in dopamine neurotransmission, if any, may be related to adolescent changes in sex steroids requires the measurement and manipulation of steroid levels across adolescence. Although there is much information from rodent studies regarding dopamine in the male adolescent period, and also many rodent studies modulating sex steroids at adulthood, there are fewer studies looking at the impact of sex steroids manipulation on dopamine signaling in adolescence. Given the developmental changes in some dopamine parameters across the lifespan, it is vital to study sex hormone-dopamine interactions in adolescence directly, rather than extrapolating from studies in preadolescent rodents and/or adult rodents [this is discussed in detail in McCutcheon and Marinelli (2009)].

The relatively few studies in clearly defined ages within the adolescent period in rodents indicate that testosterone can regulate dopamine neurotransmission. This may be particularly pertinent in schizophrenia, as males are more severely impacted (McGrath et al. 2004), and cognitive functions including working memory, processing speed, and verbal memory may be related to testosterone levels in men with schizophrenia (Moore et al. 2013). In male rats, experimental augmentation of testosterone during adolescence (between PND45 and 60) increases dopamine synthesis (Purves-Tyson et al. 2012) and stimulates midbrain expression of DR2 mRNA, dopamine transporter (DAT) mRNA, DAT protein, and vesicular monoamine transporter (VMAT) mRNA at the level of the cell body (Purves-Tyson, under review). This suggests that somatodendritic control of dopamine neurons may be most sensitive to increases in testosterone in adolescent males. At a molecular level, testosterone-induced increases in these dopamine parameters may be largely mediated via AR, rather than ER, since they are mainly mimicked by DHT (Purves-Tyson et al. 2012). Another study revealed decreased amphetamine-induced dopamine release and TH expression in the dorsal striatum in early adolescent male rats (PND34–38) compared to adult males (PND70–80) (Matthews et al. 2013). Importantly, PND34–38 is either prior to, or at the beginning of, the rise in circulating testosterone, which has been shown to be steepest between PND40 and 56 (Hill et al. 2012; Saksena and Lau 1979; Walker et al. 2012). As such, the observed increases in amphetamine-induced dopamine release and TH expression in adults (relative to younger animals) could be due to the increase in circulating testosterone. Other studies, albeit in adult male rats (although exact ages were not reported), described reductions in TH activity in the striatum, TH immunoreactive fiber density in the PFC, and extracellular dopamine levels in the PFC following gonadectomy, all of which were prevented or attenuated by testosterone replacement (Abreu et al. 1988; Aubele and Kritzer 2011; Kritzer et al. 1999). Gonadectomy of adult male rats can also increase MAOA activity in the PFC, which can be reversed by testosterone replacement (Meyers et al. 2010). Interestingly, TH immunoreactive fiber densities and extracellular dopamine levels in the PFC increase after gonadectomy to higher levels than before surgery, but this is also prevented by testosterone replacement (Aubele and Kritzer 2011; Kritzer et al. 1999). Gonadectomy-induced changes in TH immunoreactive fiber innervation and extracellular dopamine levels in the adult PFC, which are rescued by testosterone, cannot be rescued by estrogen (Aubele and Kritzer 2011; Kritzer et al. 1999). These studies indicate a major contribution by testosterone to the regulation of cortical dopamine in male rodents at the level of dopamine synthesis, transport, and metabolism. This testosterone modulation of dopamine is supported by behavioral studies, which show that, in adult male rats (PND90+), gonadectomy impairs acquisition and/or negatively impacts performance in a number of tasks including novel object recognition and behavioral flexibility (Aubele et al. 2008; Ceccarelli et al. 2001; Kritzer et al. 2007; Sandstrom et al. 2006). Studies in nonhuman primates confirm testosterone modulation of dopamine. In late-adolescent (50-month-old) male rhesus macaques, circulating testosterone was positively correlated with protein levels of TH in the striatum, while prepulse inhibition, a measure of sensory motor gating (that is regulated by dopamine and which is decreased in schizophrenia patients), was attenuated by the presence of gonadal steroids (Morris et al. 2010). In sum, although further work is required in adolescent animals specifically, it is clear that testosterone modulates many parameters of dopamine neurotransmission in the brain, both in adolescence and adulthood.

Estrogen may also play a key role in modulating dopamine neurotransmission and may contribute to adolescent changes in dopamine signaling. This is of particular interest in the context of schizophrenia, in which dopamine signaling dysfunction is implicated, initial symptoms first emerge during adolescence, females are less likely to be diagnosed than males (Hafner 2003; McGrath et al. 2004), and estrogen has been investigated as an adjunctive therapy (Akhondzadeh et al. 2003; Kulkarni et al. 2011; Kulkarni et al. 2002). Removal of circulating estrogen by ovariectomy of adult female rats (PND84) has been reported to reduce striatal DAT and increase DR2, and this can be reversed by estrogen replacement (Chavez et al. 2010). In contrast, our group found that estrogen did not modify the expression of midbrain dopamine markers in male rats following adolescent gonadectomy (between PND45 and 60) (Purves-Tyson et al. 2012). In adult (PND84) female rats, estrogen is protective against sensorimotor gating disruptions induced by DR1/DR2 agonist apomorphine, while testosterone treatment has no effect (Gogos et al. 2010; Gogos et al. 2012). In adult female primates, ovariectomy decreases TH-positive fiber density and alters fiber morphology in the PFC (Kritzer and Kohama 1998). These abnormalities are rescued by replacement of estrogen and progesterone, but not estrogen alone (Kritzer and Kohama 1998). While these results suggest that changes in sex steroids can modify dopamine neurotransmission and related behaviors in adult females, further studies are required to understand if increases in estrogen at adolescence would impact female dopamine neurons in a similar manner. Since gender differences are also a feature of neuropsychiatric illnesses including schizophrenia and depression (Hankin et al. 1998), it would be of interest to gain a more complete picture in both genders of how sex steroids and dopamine may interact during adolescence at the neurobiological level to bring about increased risk for major mental illness.

## Effects of stress on dopamine signaling

There is evidence that stress hormones can also modulate dopamine neurotransmission and dopamine-mediated cognitive function during adolescence. This is important in the context of psychiatric illness, since at-risk young people experience high levels of stress (Pruessner et al. 2011) and are more likely to develop psychosis if they have decreased tolerance of stress (Yung et al. 2005). In addition, stress can precipitate the onset of, or relapse into, psychosis (Chabungbam et al. 2007; Day et al. 1987; Myin-Germeys and van Os 2007; Norman and Malla 1993; Ventura et al. 1992). Here, we focus on stress studies in adolescent animals, but also draw attention to some work in adults which, if performed in adolescent animals, may further illuminate key aspects of the relationships between stress, glucocorticoids, and dopamine during the adolescent developmental window. Details of all studies mentioned are provided in Table 1.

**Table 1:** Studies of the effects of stress/glucocorticoids on dopamine neurotransmission

Experimental paradigm	Species (strain), gender	Age range at time of intervention	Type of intervention	Age at time of assay	Brain region(s)	Finding(s) [after stress/intervention, relative to controls]	Reference(s)
Studies in adolescents							
Postmortem brain analysis after chronic stress	Rat (SD), M/F	PND35–40	Chronic stress: 5-day social defeat	PND63	mPFC Striatum	Decreased basal dopamine Increased DAT binding Increased DR1 binding	Novick et al. (2011)
Postmortem brain analysis after chronic stress	Rat (SD), M/F	PND35–40	Chronic stress: 5-day social defeat	PND63	mPFC	Decreased basal dopamine	Watt et al. (2009)
Behavior and postmortem brain analysis after chronic stress	Rat (Long-Evans ), M/F	PND40–48	Chronic stress: 5-day intermittent exposure to predator odor	PND62	PFC Behavior	Decreased DR2 protein Hyperactivity in open field (anxiety-like)	Wright et al. (2008)
Postmortem brain analysis after chronic stress plus amphetamine	Rat (SD), M/F	PND35–40	Chronic stress: 5-day social defeat	PND63	mPFC Striatum	Decreased basal dopamine Increased DAT binding Increased DR1 binding	Burke et al. (2010, 2011, 2013)
Behavior and postmortem brain analysis after chronic stress	Rat (Wistar Han), M/F	PND28–42	Chronic stress: 7-day intermittent predator odor and aversive environment	PND104–125	mPFC Behavior	Increased MAOA gene expression Increased MAOA promoter histone H3 acetylation Increased aggression	Marquez et al. (2013)
Behavior and electrophysiology after chronic stress	Rat (Long-Evans), M/F	PND28–77	Chronic stress: 6-week social isolation	Immediately after chronic stress (PND77)	NAc Behavior	Increased electrically induced dopamine release and uptake Decreased time on open arm in elevated plus maze	Yorgason et al. (2013)
Electrophysiology, behavior, and postmortem brain analysis after chronic stress	DISC1 (dominant-negative) mutant mouse, M/F	PND21–56	Chronic stress: 3-week isolation stress	Immediately after chronic stress (PND56)	HPA axis PFC^#^ Behavior^#^	Increased stress-induced corticosterone secretion Decreased basal dopamine Increased TH gene methylation Impaired PPI Depression/anxiety-like behaviors	Niwa et al. (2013)
Postmortem brain immunohistochemistry after chronic stress	Rat (SD), M/F	PND51–58 (M) PND55–62 (F)	Chronic stress: 7-day restraint stress	Immediately after chronic stress (PND58–62)	mPFC	M and F(OVX): decreased apical dendrite length F and F(OVX + E): increased apical dendrite length	Garrett and Wellman (2009)
Studies in late adolescents/young adults and adults							
Live imaging of DR2/3 binding using PET ([^18^ F]fallypride) during acute stress	Human, M	18–30 (mean 22.6)	Acute stress: psychosocial	During acute stress	mPFC	Increased dopamine release during stress	Nagano-Saito et al. (2013)
fMRI during acute stress	Human, F	18–25 (mean 22.6)	Acute stress: psychosocial stress during reward task	During acute stress	mPFC	Decreased reward-related mPFC activation during stress	Ossewaarde et al. (2011)
fMRI during acute stress	Human, M	18–25	Acute stress during working memory task	During acute stress	DLPFC	Decreased working memory-related DLPFC activation during stress	Qin et al. (2009)
Live imaging of DR2/3 binding using PET ([^11^C]raclopride)	Human, M/F	18–35	Acute stress: psychosocial	During acute stress	Striatum	Increased dopamine release during stress	Pruessner et al. (2004)
Live imaging of DR2/3 binding using PET ([^11^C]raclopride)	Human, M/F	18–29	Acute stress: psychosocial	During acute stress	Striatum	Increased amphetamine-induced dopamine release during stress	Wand et al. (2007)
Live imaging of DR2/3 binding using PET ([^11^C]raclopride)	Human, M/F	21–31	Acute stress: psychosocial	During acute stress	Striatum	Leptin genotype influences dopamine release during stress	Burghardt et al. (2012)
Selected studies in adults							
In vivo reverse microdialysis during acute stress or PFC corticosterone injection, with/without GR antagonist	Rat (SD), M	PND60–70^	Acute stress: mild tail pinch	During acute stress	PFC	Increased dopamine efflux after stress. Attenuated stress-or corticosterone-induced PFC dopamine efflux after administration of GR antagonist RU38486 into the PFC, but not the VTA	Butts et al. (2011)
In vivo microdialysis after acute stress	Rat (Wistar), M/F	PND60–90	Acute stress: 60 min restraint stress	PND60–90	Amygdala	Increased amygdala dopamine levels after acute stress in females, but not males	Mitsushima et al. (2006)
In vivo microdialysis and adrenalectomy	Rat (SD)	PND90^	Basal levels and acute mild stress (sham injection)	PND90^	NAc (shell)	Decreased dopamine release in ADX rats relative to controls and ADX + corticosterone rats	Barrot et al. (2000), Piazza et al. (1996)
Postmortem brain analysis after chronic stress	Rat (Wistar)	PND85^	Chronic stress	PND100^	Striatum, NAc, VTA	Decreased DR2 density 14 days after stress, but no change in DR2 mRNA. DR2 deficit sustained (>35 days) in stress-sensitive rats, but transient (<35 days) in resilient rats	Zurawek et al. (2013)
Wild-type and mutant mice with selective GR inactivation in dopaminergic and dopaminoceptive neurons, plus chronic stress	Mouse, M	PND60–120	Chronic stress: 10-day social defeat	Immediately after chronic stress	VTA NAc Behavior PFC, striatum, NAc	Increased firing of dopamine neurons in WT mice Increased dopamine release in WT mice Social aversion, rescued by treatment with quinpirole (DR2 agonist that suppresses dopamine neuron activity) Abolition of above effects by selective inactivation of GR in dopamine-responsive neurons of the cortex (layers V and VI only), striatum, and NAc	Barik et al. (2013)

*Abbreviations*: *PND* postnatal day, *M* male, *F* female, *SD* Sprague-Dawley, (*m*)*PFC* (medial) prefrontal cortex, *DLPFC* dorsolateral prefrontal cortex, *NAc* nucleus accumbens, *SN* substantia nigra, *VTA* ventral tegmental area, *GR* glucocorticoid receptor, *DR* dopamine receptor, *DAT* dopamine transporter, *MAOA* monoamine oxidase A, *TH* tyrosine hydroxylase, *PPI* prepulse inhibition, *WT* wild type, *ADX* adrenalectomized, *OVX* ovariectomized, *E* estrogen, *PET* positron emission tomography, *fMRI* functional magnetic resonance imaging, # observations made in DISC1 mutant mice but not WT littermates, ^studies in which animal weights only were published, but study authors were contacted for exact animal ages

Acute stress in late adolescence/early adulthood has been shown to trigger dopamine release in the brain. In humans (ages 18–30), dopamine release occurs in the PFC in response to psychological stress (Nagano-Saito et al. 2013), in parallel with decreased working memory-related and reward-related PFC activation (Ossewaarde et al. 2011; Qin et al. 2009) and impaired working memory performance in males (Schoofs et al. 2013). This suggests that increased PFC dopamine secretion may impair, rather than facilitate, PFC function during adolescent stress. Dopamine release can also occur in the striatum in response to acute psychosocial stress and after stress in the presence of amphetamine, in individuals aged 18–35 (Burghardt et al. 2012; Pruessner et al. 2004; Wand et al. 2007). This stress-induced dopamine increase is positively correlated with the magnitude of salivary cortisol response (Pruessner et al. 2004). These findings are consistent with the adult rodent literature, which support the involvement of glucocorticoids in modulating stress-induced dopamine release (Table 1). Interestingly, individuals with schizophrenia, individuals at clinical high risk of schizophrenia, and individuals with negative prodromal symptoms (all aged 18–35) display greater striatal dopamine release in response to stress than controls (Mizrahi et al. 2012; Soliman et al. 2008). It is worth noting that immediate stress-induced dopamine secretion may be influenced not only by glucocorticoids, but also by inputs from brain circuitry evaluating stressors (Joels and Baram 2009; Ulrich-Lai and Herman 2009). Caution in interpreting the relevance to adolescence of the findings above is warranted, since these studies included both individuals aged 18–25 (who may be considered late adolescent/young adult) and individuals aged 25–35 (adult). However, the rapid modulation of dopamine in the brain by a single stress exposure suggests that acutely stressful experiences in adolescence may have an immediate impact on brain function and behavior at key moments in an adolescent’s life.

Chronic stress during adolescence has long-lasting, deleterious effects on dopamine function and dopamine-related behaviors. Experimental paradigms used to examine the effects of chronic stress, particularly in rodents, have commonly employed social defeat (repeated exposure to a dominant aggressor), isolation, restraint, or exposure to aversive odors or environments (see Table 1). Five- to 7-day social defeat of rats between PND28 and 48 induces long-term dopamine-related changes in the PFC in adulthood, such as decreased basal dopamine levels, decreased DR2 expression, increased DAT binding, increased MAOA gene expression, and increased MAOA promoter histone acetylation (Marquez et al. 2013; Novick et al. 2011; Watt et al. 2009; Wright et al. 2008). Also evident are changes in the striatum, such as increased DR1 binding (Novick et al. 2011), and abnormal behavior, such as increased aggression and anxiety-like behaviors (Marquez et al. 2013; Wright et al. 2008). Chronic social defeat in adolescence (PND35–40) also alters responses to amphetamine, resulting in increased locomotion, decreased corticosterone secretion, decreased medial PFC dopamine levels, increased NAc core dopamine levels, and impaired DR2 downregulation in the NAc core (Burke et al. 2010, 2011, 2013). These findings suggest that chronic stress during adolescence may detrimentally impact the developmental trajectory of dopaminergic circuits, leading to long-term molecular and behavioral maladaptations.

The mechanisms underlying the effects of chronic stress on dopaminergic neurotransmission in adolescence have been illuminated by a small number of studies in adolescent animals, with additional clues provided by studies in adults. In late-adolescent rats, 7 days of chronic restraint stress (between PND51 and 58 in males and between PND55 and 62 in females) has gender-specific effects on apical dendritic length of pyramidal neurons in layers II–III in the medial PFC (Garrett and Wellman 2009). Males display decreased apical dendritic length after stress, while females display increased apical dendritic length which can be ameliorated by ovariectomy and restored by ovariectomy combined with estrogen replacement (Garrett and Wellman 2009). These findings suggest that stress may induce changes in neuronal morphology in the PFC, potentially impacting available target sites for incoming dopamine afferents, and that these effects may be modulated also by sex hormones. Chronic stress effects on dopamine neurotransmission may also arise from control of dopamine-related gene transcription by GR in key brain regions. For example, MAOA is a GR target gene whose expression is rapidly increased by glucocorticoid administration in the adolescent rat hippocampus (PND42) (Morsink et al. 2006) and is persistently increased, following adolescent chronic stress (PND28–42), in the adult rat PFC (PND104–125) (Marquez et al. 2013). Two additional noteworthy studies have been conducted in young adult/adult rodents. Firstly, administration of the GR antagonist RU38486 into the PFC, but not the VTA, has been shown to attenuate acute stress-induced dopamine efflux in the PFC, and working memory impairment, in young adult rats (PND60–70) (Butts et al. 2011). This highlights a potential role for GR, specifically within dopamine-responsive neurons in dopamine neuron projection areas, in mediating the effects of stress on dopamine signaling and cognition in young adult rats. These findings were supported by a detailed study in adult (PND60–120) mice, which demonstrated that selective ablation of GR in dopamine-responsive neurons of the striatum, NAc, and cortex (layers V and VI only) abolishes the effects of chronic stress on social behaviors, eliminates stress-induced increases in dopamine neuron firing in the VTA, and diminishes the stress-induced increase in dopamine release in the NAc (Barik et al. 2013). Ablation of GR in dopamine neurons of the SN and VTA has no such effects (Barik et al. 2013). These approaches could be extended to adolescent animals, to determine whether the adolescent effects of chronic stress on dopamine signaling are mediated by GR, specifically in dopamine-responsive neurons rather than in dopamine neurons themselves. A detailed understanding of which cell populations and molecular mechanisms mediate the effects of stress on dopamine signaling in adolescence may lead to future studies aiming to ameliorate these effects. Ultimately, such studies could identify targets for interventions to buffer the deleterious effects of stress in vulnerable individuals, particularly in the context of illnesses such as schizophrenia in which dopamine signaling has been implicated.

## Conclusion

While adolescence has long been recognized as a key time of behavioral change in animals and humans, the neurobiological underpinnings of these changes are just beginning to be characterized. The scarcity of information about the major neurobiological changes occurring in adolescence limits our ability to understand vulnerabilities that may increase the risk for developing major mental illness during this time. Evidence is emerging that adolescence is a time of heightened responsiveness to stress and sex hormones and that these hormones can interact to modulate each other’s function at adolescence. Furthermore, the maturation and function of dopamine neurotransmission in cortical and subcortical brain regions can be impacted at adolescence by sex and stress hormones, in a brain region and hormone-specific manner (summarized in Fig. 3). Sex hormones exert at least some of their effects on dopamine signaling through midbrain dopamine neurons, by acting directly and indirectly on dopamine cell bodies to control changes in dopamine synthesis and response during adolescent brain maturation. Interestingly, if stress hormones act by similar mechanisms on dopamine signaling in adolescence as in adulthood, then dopaminoceptive neurons in the PFC, striatum, and NAc, rather than midbrain dopaminergic neurons, may be the targets of stress hormones. Our current review emphasizes that sex and stress hormones work together to tune dopamine responses in the human brain during adolescent maturation. Further, adolescent hormones may have different actions on dopamine in males and females, which may be distinct from hormone actions in adult brains.

Clarifying sex and stress hormone effects on dopamine neurotransmission may aid understanding of the possible roles of these hormones in dopamine-related psychiatric disorders. For comparison purposes, some dopamine-related abnormalities which are found in schizophrenia are presented alongside the effects of sex and stress hormones on dopamine neurotransmission in Fig. 3. As an example, since dopamine release in the striatum increases progressively during the transition to psychosis in schizophrenia (Howes et al. 2011; Howes et al. 2009), and acute stress has been shown to increase striatal dopamine release in young adults at high risk of schizophrenia (Mizrahi et al. 2012; Pruessner et al. 2004), it is plausible that acute stress in young adults at risk of schizophrenia may worsen underlying striatal hyperdopaminergia to increase prodromal symptoms or risk of psychosis. Moderating the effects of stress on dopamine signaling in vulnerable individuals may therefore help reduce the risk of transition to psychosis around adolescence. In addition, the development of pharmacological interventions which exploit the effects of sex hormones on dopamine signaling may enable tailored therapies to restore neurotransmitter balance in key brain areas related to the pathophysiology of these illnesses. For example, inhibition of 5α reductase, which converts testosterone to the more potent androgen DHT, has been proposed as a novel antipsychotic (Paba et al. 2011), while estrogen therapies have shown promise as adjunctives to antipsychotics (Kulkarni et al. 2002). Ultimately, changes in dopamine neurotransmission must be understood in the context of the complex changes in other neurotransmitter systems during adolescence (Catts et al. 2013), which represents a challenge for future studies.
